# Assessing quality assurance of multi‐leaf collimator using the structural similarity index

**DOI:** 10.1002/acm2.14288

**Published:** 2024-02-12

**Authors:** Hong Zhang, Baoshe Zhang, Giovanni Lasio, Shifeng Chen, Joubin Nasehi Tehrani

**Affiliations:** ^1^ Departments of Radiation Oncology Vanderbilt University Medical Center Nashville Tennessee USA; ^2^ Departments of Radiation Oncology Medical School University of Maryland Baltimore Maryland USA

**Keywords:** Gamma index test, MLC, QA, SSI*, Structural Similarity Index, VMAT

## Abstract

**Purpose:**

This study aims to evaluate the viability of utilizing the Structural Similarity Index (SSI*) as an innovative imaging metric for quality assurance (QA) of the multi‐leaf collimator (MLC). Additionally, we compared the results obtained through SSI* with those derived from a conventional Gamma index test for three types of Varian machines (Trilogy, Truebeam, and Edge) over a 12‐week period of MLC QA in our clinic.

**Method:**

To assess sensitivity to MLC positioning errors, we designed a 1 cm slit on the reference MLC, subsequently shifted by 0.5–5 mm on the target MLC. For evaluating sensitivity to output error, we irradiated five 25 cm × 25 cm open fields on the portal image with varying Monitor Units (MUs) of 96–100. We compared SSI* and Gamma index tests using three linear accelerator (LINAC) machines: Varian Trilogy, Truebeam, and Edge, with MLC leaf widths of 1, 0.5, and 0.25 mm. Weekly QA included VMAT and static field modes, with Picket fence test images acquired. Mechanical uncertainties related to the LINAC head, electronic portal imaging device (EPID), and MLC during gantry rotation and leaf motion were monitored.

**Results:**

The Gamma index test started detecting the MLC shift at a threshold of 4 mm, whereas the SSI* metric showed sensitivity to shifts as small as 2 mm. Moreover, the Gamma index test identified dose changes at 95MUs, indicating a 5% dose difference based on the distance to agreement (DTA)/dose difference (DD) criteria of 1 mm/3%. In contrast, the SSI* metric alerted to dose differences starting from 97MUs, corresponding to a 3% dose difference. The Gamma index test passed all measurements conducted on each machine. However, the SSI* metric rejected all measurements from the Edge and Trilogy machines and two from the Truebeam.

**Conclusions:**

Our findings demonstrate that the SSI* exhibits greater sensitivity than the Gamma index test in detecting MLC positioning errors and dose changes between static and VMAT modes. The SSI* metric outperformed the Gamma index test regarding sensitivity across these parameters.

## 1 INTRODUCTION

Radiation therapy is a widely recognized and effective cancer treatment method focusing on delivering radiation precisely to the tumor while minimizing harm to adjacent healthy tissues. To accomplish this, multi‐leaf collimators (MLC) have become an integral component of modern radiation therapy delivery techniques.^1^ Typically constructed with parallel leaves made of high atomic number materials like tungsten, the MLC plays a crucial role in shaping the radiation field and achieving a conformal and customized fit to the target area.^2^ By utilizing each movable leaf independently, the MLC can block over 90% of the radiation, enabling modulation of the radiation field to precisely administer the prescribed dose to the designated target volume.^3^, ^4^

**Table:** 

The clinical data used in section 3.4 were derived from public domain resources	Data citation Wee, L., Aerts, H. J.L., Kalendralis, P., & Dekker, A. (2019). **Data from NSCLC‐Radiomics‐Interobserver1 [Data set]**. The Cancer Imaging Archive. https://doi.org/10.7937/tcia.2019.cwvlpd26. TCIA citation Clark K, Vendt B, Smith K, Freymann J, Kirby J, Koppel P, Moore S, Phillips S, Maffitt D, Pringle M, Tarbox L, Prior F. **The Cancer Imaging Archive (TCIA): Maintaining and Operating a Public Information Repository**, Journal of Digital Imaging, Volume 26, Number 6, December, 2013, pp 1045−1057. DOI: 10.1007/s10278‐013‐9622‐7 https://wiki.cancerimagingarchive.net/display/Public/NSCLC‐Radiomics‐Interobserver1Case#5

MLC has emerged as a critical device in modern radiation therapy (RT), particularly with the advent of intensity‐modulated radiotherapy (IMRT) and volumetric‐modulated arc therapy (VMAT).^5^ These advanced techniques have demonstrated advantages over conventional RT in minimizing radiation dose to healthy tissues. IMRT and VMAT utilize inverse planning methods to vary MLC shapes, gantry rotation speed, and dose rate, enabling the precise delivery of the intended dose distribution.^6^

The accurate positioning and motion of the multi‐leaf collimator (MLC) are of utmost importance in ensuring precise and effective radiation treatment, and therefore, regular quality assurance (QA) is essential.^7^ One significant factor that can influence MLC positioning errors is the effect of gravity on the mechanical components of the gantry head, particularly evident during VMAT tests.^8^ Gravity‐induced wobbling can occur in the gantry and the MLC, leading to a degradation in the delivered dose distribution.^9^ To address this issue, our institution conducts weekly QA assessments by comparing dose matches between a VMAT plan and the same plan utilizing a static‐gantry‐sliding window technique.^10^

Traditionally, the Gamma index test has been a widely used method for evaluating the accuracy of radiation delivery.^1^, ^11^, ^12^, ^13^ The Gamma index test, commonly utilized in radiation therapy, compares the agreement between planned and measured dose distributions. It employs predefined acceptance criteria to determine whether radiation delivery meets the desired accuracy standards. However, it is essential to acknowledge that this method has limitations when detecting tiny gaps in MLC positioning and dose output. These errors, despite being subtle, can significantly impact treatment quality.^14^, ^15^, ^16^

To address the limitations of the Gamma index test, the structural similarity index (SSI) has recently emerged as a promising new imaging metric for weekly quality assurance (QA) of MLCs.^17^, ^18^ The SSI evaluates image quality by assessing the similarity between images, such as the portal and reference images, based on luminance, contrast, and structural information.^19^, ^20^ This approach has demonstrated increased sensitivity to subtle MLC errors, potentially enhancing the accuracy of MLC QA.^21^

In our study, we implemented a picket fence plan using two different delivery techniques: static and volumetric modulated arc therapy (VMAT).^22^ We subsequently compared the two‐dose maps obtained on the electronic portal imaging device (EPID) to identify any deviations from the distinct delivery methods.^23^ We employed the conventional Gamma index test as the primary method for comparing the dose maps obtained from the static and VMAT delivery techniques. We introduced the Structural Similarity Index (SSI) as a secondary method to evaluate its sensitivity in detecting deviations, in comparison to the Gamma index. To enhance the sensitivity of SSI, we implemented a scaling approach by adjusting the luminance component to enable detection of even small intensity differences, specifically aiming for a 3% threshold.

## 2 MATERIALS AND METHODS

### 2.1 Dataset collection

We conducted tests on three types of Varian linear accelerators, Trilogy, Truebeam, and Edge—using MLCs of varying sizes and leaf widths. Specifically, the Millennium 120 MLC, installed on Trilogy and Truebeam machines, featured 120 leaves. Among these leaves, the central 80 had a projection leaf width of 0.5 cm, while the outer 40 leaves had a projection leaf width of 1.0 cm at the isocenter.^24^, ^25^ The Edge linear accelerator utilizes a high definition 120 multi‐leaf collimator (MLC) with 120 leaves as well. The MLC configuration consists of 64 central leaves with a projection leaf width of 0.25 cm at the isocenter, while the remaining 56 outer leaves have a projection leaf width of 0.5 cm.^26^ In our investigation, we conducted a comprehensive 12‐week comparative analysis utilizing two distinct evaluation methods: the Gamma index test and an enhanced variant of the SSI, denoted as SSI* in this paper. The delivery approach encompassed both a static and a VMAT technique employing the picket fence pattern. Furthermore, a VMAT plan was generated for lung treatment, based on a real patient's CT scan data. To explore variations, the MLC was adjusted for each control point by increments of 0.5, 1, 3, and 5 mm. Subsequently, both the original plan and the four variants with diverse MLC shifts were administered using a Truebeam machine. The beam energy for all tests was 6MV, and the MLC type was the Millennium 120 MLC.

### 2.2 Gamma index test

We can name the static map as $D_{s}\left(\vec{r_{s}}\right)$ and the VMAT map as $D_{v}\left(\vec{r_{v}}\right)$ where D is the dose and $\vec{r}$ is the position. The Gamma index used in the test is calculated as^13^, ^27^, ^28^

(1) $$\Gamma \left(\vec{r_{s}},\vec{r_{v}}\right)=\sqrt{\frac{r^{2}\left(\vec{r_{s}},\vec{r_{v}}\right)}{{\left(\Delta d\right)}^{2}}+\frac{\delta^{2}\left(\vec{r_{s}},\vec{r_{v}}\right)}{{\left(\Delta D\right)}^{2}}},$$

where $r\left(\vec{r_{s}},\vec{r_{v}}\right)=\left|\left(\vec{r_{s}}-\vec{r_{v}}\right)\right|$, is the spatial Distance‐to‐Agreement (DTA) distance between the static and VMAT positions, $\delta \left(\vec{r_{s}},\vec{r_{v}}\right)=D_{s}\left(\vec{r_{s}}\right)-D_{v}\left(\vec{r_{v}}\right)$, is the dose difference between the two positions, Δd is the acceptance DTA criterion, and ΔD is the acceptance dose difference criterion. The distance to agreement (DTA) is a measure that quantifies the spatial difference between a point in the static dose distribution and the closest corresponding point in the VMAT dose distribution, where both points share the same dose value. It is defined as^29^:

(2) $$\begin{array}{ccc} DTA\left(\vec{r_{s}}\right) & = & min\left\{\left|\vec{r_{s}}-\vec{r_{v}}\right|\right\},\forall \left\{\vec{r_{s}}\right\},\\ & & where\;\;D_{s}\left(\vec{r_{s}}\right)=D_{v}\left(\vec{r_{v}}\right) \end{array}$$

When $\Gamma (\vec{r_{s}},\vec{r_{v}})\leq 1$, the test for this point passes, and when $\Gamma (\vec{r_{s}},\vec{r_{v}})>1$, the test for this point fails. Often Δd is set to 3 mm, ΔD is set to 3% of the dose, and if 90% of points within the dose distribution under analysis pass the Gamma index test, the conditions for passing the QA are considered met.

### 2.3 Structural similarity

The SSI index is another method of comparing the similarity of two 2D images.^30^ The SSI formula is based on three comparison measurements between the samples of x and y based on luminance (l), contrast (c) and structure (s).^31^ It is defined as:

(3) $$SSIM\left(x,y\right)=l{\left(x,y\right)}^{\alpha }c{\left(x,y\right)}^{\beta }s{\left(x,y\right)}^{\gamma }$$

where *l(x,y), c(x,y), s(x,y)* are luminance, contrast and structure, respectively, and are defined as:

(4) $$l\left(x,y\right)=\frac{2\mu_{x}\mu_{y}+C_{1}}{\mu_{x}^{2}+\mu_{y}^{2}+C_{1}}$$

(5) $$c\left(x,y\right)=\frac{2\sigma_{x}\sigma_{y}+C_{2}}{\sigma_{x}^{2}+\sigma_{y}^{2}+C_{2}}$$

(6) $$s\left(x,y\right)=\frac{\sigma_{xy}+C_{3}}{\sigma_{x}\sigma_{y}+C_{3}}$$

and $C_{1}$, $C_{2}$, and $C_{3}$ are regularisation constants to avoid small denominators. Based on the literature, we set α=β=γ=1 and $C_{3}=C_{2}/2$. Here $\mu_{x}$ and $\mu_{y}$, $\sigma_{x}$, $\sigma_{y}$, and $\sigma_{xy}$ are the local means, standard deviations and cross‐covariance of image X and image Y.

(7) $$\mu_{x}=\frac{1}{N}\sum_{i\;=\;1}^{N}x_{i}$$

(8) $$\sigma_{x}={\left(\frac{1}{N-1}\sum_{i=1}^{N}{\left(x_{i}-\mu_{x}\right)}^{2}\right)}^{0.5}$$

(9) $$\mu_{xy}=\frac{1}{N-1}\sum_{i\;=\;1}^{N}\left(x_{i}-\mu_{x}\right)\left(y_{i}-\mu_{y}\right)$$

where *i* is the index of points inside a local sampling window of N=w×w pixels.

Luminance represents the dose difference, and contrast and structure represent the MLC position difference, in our application.

For this reason, SSI is simplified to:

(10) $$\begin{array}{ccc} SSIM\left(x,y\right) & = & l\left(x,y\right)C\_S\left(x,y\right)\\ & & \;\;\;\;\;\;=\frac{\left(2\mu_{x}\mu_{y}+C_{1}\right)\left(2\sigma_{xy}+C_{2}\right)}{\left(\mu_{x}^{2}+\mu_{y}^{2}+C_{1}\right)\left(\sigma_{x}^{2}+\sigma_{y}^{2}+C_{2}\right)} \end{array}$$

where the luminance component remains as in (4) and *C_S* is:

(11) $$C\_S\left(x,y\right)=\frac{2\sigma_{xy}+C_{2}}{\sigma_{x}^{2}+\sigma_{y}^{2}+C_{2}}$$

### 2.4 Revision of luminance component

**FIGURE 1:**
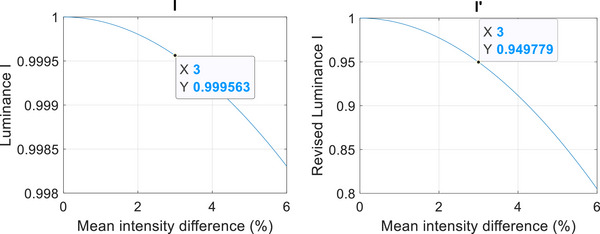
Left shows SSI luminance curve and right shows SSI* luminance curve.

In our application, the luminance component of the Structural Similarity Index (SSI) is utilized to identify dose (i.e. intensity) changes between two images. However, we have observed that this component lacks the required sensitivity for accurate intensity comparison in our specific output evaluation scenario, as demonstrated in Figure 1 left.

For instance, when the mean intensity difference between the images is 3%, the corresponding change in the luminance component is only 0.999563. This small change is insufficient to reliably detect and assess the desired intensity variation.

To overcome the sensitivity limitation of the luminance component (l) in detecting intensity changes, we propose enhancing its sensitivity by modifying it to $l^{\prime }\;=\;1-115\left(1-l\right)$ this modification preserves the same functional shape (Figure 1 Right). This adjustment aims to improve the luminance component's ability to accurately capture and quantify even subtle variations in intensity. The parameter of 115 is set so that by scaling *l* to make the *l'* drop to 0.95 when the mean intensity difference is 3%, which is a difference we expect to detect. The revised SSI*(x, y) is defined as $l^{\prime }\left(x,y\right)C\_S\left(x,y\right)$.

### 2.5 Comparison

**FIGURE 2:**
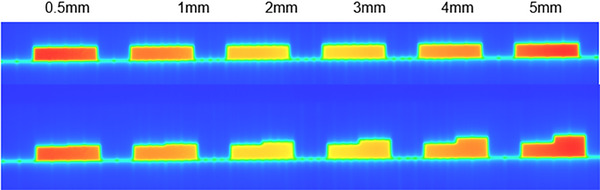
The slits with different shifts (0.5, 1, 2, 3, 4, 5 mm). The above is the reference portal image, and below is the target portal image.

In this study, we performed the Gamma index test and SSI* analysis using different MLCs over sequential weeks. The goal was to compare the performance of these two methods and identify their respective advantages and disadvantages. To evaluate the sensitivity of the two methods to MLC positioning errors, we planned a 1 cm slit as a reference MLC and then shifted the slit by 0.5, 1, 2, 3, 4, and 5 mm on the target MLC, as shown in Figure 2. Two portal images were acquired using an electronic portal image device (EPID) for each MLC pattern.^32^

For each shift, we performed the Gamma index test using both 3 mm/3% and 1 mm/3% criteria, as well as the SSI* analysis with sampling window sizes of 3 × 3 and 5 × 5. Since the Edge machine is primarily used for SRS (Stereotactic Radiosurgery) treatments, which require highly precise and accurate delivery of radiation to small lesions or tumors, it is essential to employ a tighter tolerance level for quality assurance.^33^

By setting a more stringent passing criterion of 1 mm/3% for the Gamma index test in Edge SRS QA, you can ensure a finer evaluation of treatment accuracy. This level of tolerance allows detection of even smaller deviations from the intended dose distribution or spatial positioning, thereby enhancing the overall quality assurance process. The obtained results from these tests were subsequently compared to evaluate their respective performances.

In order to evaluate the sensitivity of the two methods to output errors, we irradiated a 25 cm × 25 cm open field on the portal image using different Monitor Units (MU) values (96, 97, 98, and 99).^34^ The image obtained with 100 MU was used as the reference image.

We also collected and compared MLC picket fence images with three strips, delivered via static field and VMAT field, over a span of 12 weeks. These measurements were conducted on Trilogy, Truebeam, and Edge machines.

## 3 RESULTS

### 3.1 Sensitivity to the position shift

The acquired reference and target images for slits with different shifts are depicted in Figure 2. Table 1 presents the results of the Gamma index test (using 3 mm/3% and 1 mm/3% criteria) and SSI* analysis (with 3 × 3 and 5 × 5 sampling windows) for each MLC shift.

**TABLE 1:** Positioning shift (red numbers represent failure cases).

Shift	0.5 mm	1 mm	2 mm	3 mm	4 mm	5 mm
Gamma index (3 mm/3%)	98.7	99.5	99.9	98.6	96.9	95.7
Gamma index (1 mm/3%)	96.3	97.2	97.2	96.2	94.8	93.4
l' (3 × 3)	0.999	0.998	0.996	0.992	0.984	0.971
*C_S* (3 × 3)	0.976	0.965	0.937	0.909	0.884	0.864
SSI* (3 × 3)	0.975	0.963	0.934	0.902	0.87	0.839
l' (5 × 5)	0.999	0.999	0.997	0.992	0.985	0.973
*C_S* (5 × 5)	0.976	0.964	0.932	0.9	0.869	0.845
SSI* (5 × 5)	0.975	0.963	0.929	0.893	0.856	0.822

In the tests we performed we define a failure case as an index (Gamma or SSI*) value that is less than 0.95.

It was observed that the Gamma index test started to detect the shift from 4 mm, whereas SSI* detected a 2 mm shift. This finding highlights the higher sensitivity of SSI* compared to the Gamma index test in detecting MLC positioning errors.

### 3.2 Sensitivity to dose change

For different MUs, the Gamma index test (3 mm/3% and 1 mm/3%) and SSI*’s results with 3 × 3 sampling window were listed in Table 2. It was observed that the Gamma test started to detect the dose change from 95MUs, which means a 5% dose difference with strict 1 mm/3% criteria, while SSI* alerted the dose difference from 97MUs, representing a 3% dose difference. These findings indicate that SSI* is more sensitive to detecting dose changes compared to the Gamma index test.

**TABLE 2:** Dose change (red numbers represent failure cases).

MU	99	98	97	96	95
Gamma index (3 mm/3%)	98.7	99.5	99.9	98.6	96.9
Gamma index (1 mm/3%)	96.3	97.2	97.2	96.2	94.8
l' (3 × 3)	0.994	0.977	0.947	0.905	0.851
*C_S* (3 × 3)	1	1	1	1	1
SSI* (3 × 3)	0.994	0.977	0.947	0.905	0.851

### 3.3 Sensitivity to static versus VMAT dose deviations

Tables 3, 4, 5 present the results of the Gamma index test with passing criteria set at 3 mm/3% for Trilogy and Truebeam, and 1 mm/3% for Edge, along with the SSI* results using 3 × 3 and 5 × 5 sampling windows. The Gamma index test consistently passed on each machine every week. However, the SSI* analysis revealed failures for Edge (with both sampling window sizes) on a daily basis, Trilogy (with the 5 × 5 sampling window) every week, and Truebeam for two weeks. These results indicate that the SSI* metric is more sensitive to deviations compared to the Gamma index test. In the discussion section, we present in detail one of the failure instances from Edge, where SSI* successfully detected a significant deviation that went unnoticed by the Gamma index test.

**TABLE 3:** Edge weekly MLC QA (red numbers represent failure cases).

Week	1	2	3	4	5	6	7	8	9	10	11	12
Gamma index (1 mm/3%)	98.2	98.6	98.8	98.6	98.5	98.4	98.5	98.9	98.6	98.7	98.6	98.5
*l'* (3 × 3)	0.931	0.908	0.885	0.92	0.908	0.701	0.828	0.816	0.885	0.828	0.793	0.851
*C_S* (3 × 3)	0.98	0.98	0.98	0.98	0.98	0.98	0.98	0.98	0.98	0.98	0.98	0.98
SSI*(3 × 3)	0.913	0.89	0.867	0.9	0.89	0.686	0.811	0.8	0.868	0.811	0.777	0.831
*l'* (5 × 5)	0.943	0.92	0.9	0.931	0.931	0.724	0.839	0.839	0.897	0.851	0.816	0.862
*C_S* (5 × 5)	0.978	0.977	0.977	0.977	0.979	0.977	0.977	0.978	0.978	0.977	0.977	0.975
SSI* (5 × 5)	0.922	0.898	0.876	0.91	0.911	0.707	0.82	0.821	0.877	0.831	0.797	0.84

**TABLE 4:** Trilogy weekly MLC QA (red numbers represent failure cases).

Week	1	2	3	4	5	6	7	8	9	10	11	12
Gamma index (3 mm/3%)	99.8	99.8	99.8	99.8	99.9	99.8	99.8	99.9	97.5	99.9	99.9	99.9
*l'* (3 × 3)	0.995	0.994	0.995	0.995	0.995	0.995	0.995	0.995	0.994	0.995	0.995	0.994
*C_S* (3 × 3)	0.954	0.951	0.955	0.953	0.956	0.958	0.954	0.954	0.917	0.955	0.954	0.955
SSI*(3 × 3)	0.949	0.946	0.950	0.948	0.950	0.953	0.950	0.950	0.912	0.950	0.949	0.950
*l'* (5 × 5)	0.995	0.995	0.995	0.995	0.995	0.995	0.995	0.994	0.994	0.995	0.995	0.994
*C_S* (5 × 5)	0.943	0.940	0.944	0.943	0.946	0.948	0.944	0.944	0.893	0.945	0.944	0.945
SSI* (5 × 5)	0.938	0.935	0.939	0.938	0.940	0.943	0.939	0.938	0.887	0.940	0.939	0.940

**TABLE 5:** Truebeam weekly MLC QA (red numbers represent failure cases).

Week	1	2	3	4	5	6	7	8	9	10	11	12
Gamma index (3 mm/3%)	100	100	100	100	100	100	100	100	100	100	100	100
*l'* (3 × 3)	0.954	0.954	0.954	0.954	0.966	0.943	0.966	0.954	0.954	0.920	0.954	0.954
*C_S* (3 × 3)	0.999	0.999	0.999	0.999	0.999	0.999	0.999	0.999	0.999	0.999	0.999	0.999
SSI*(3 × 3)	0.953	0.953	0.953	0.953	0.965	0.942	0.965	0.953	0.953	0.912	0.953	0.953
*l'* (5 × 5)	0.954	0.954	0.954	0.954	0.965	0.942	0.966	0.954	0.954	0.920	0.954	0.954
*C_S* (5 × 5)	0.999	0.999	0.999	0.999	0.999	0.999	0.999	0.999	0.999	0.999	0.999	0.999
SSI* (5 × 5)	0.953	0.953	0.953	0.953	0.965	0.942	0.965	0.953	0.953	0.919	0.953	0.953

### 3.4 Study of the MLC error in real plan

**FIGURE 3:**
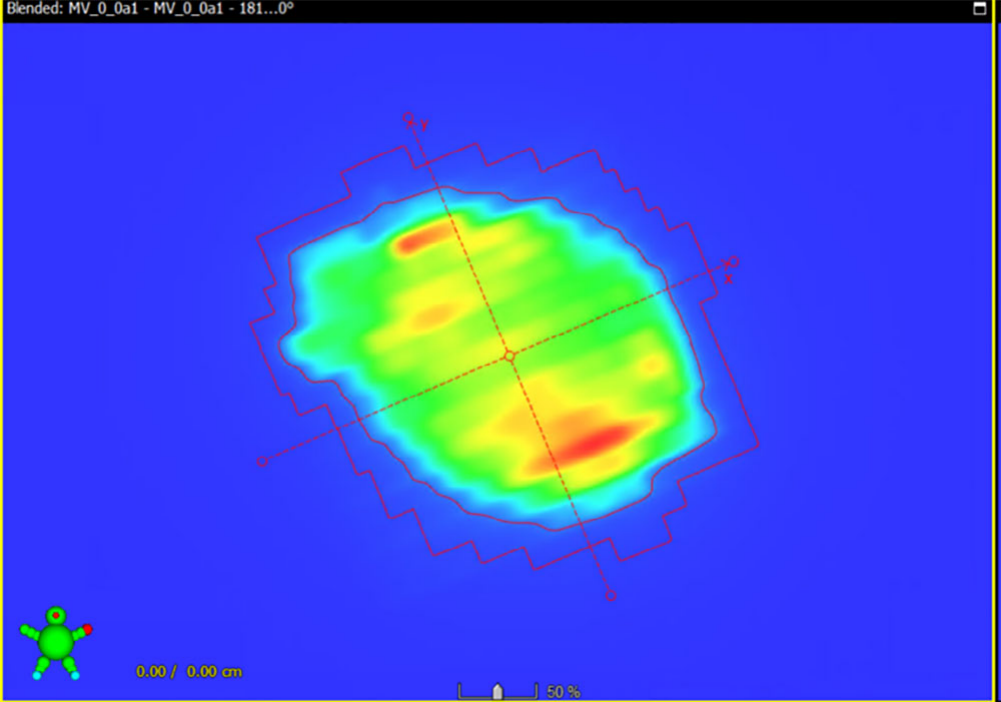
Illustration of the original (no shifted) fluence‐map of the lung treatment plan.

**FIGURE 4:**
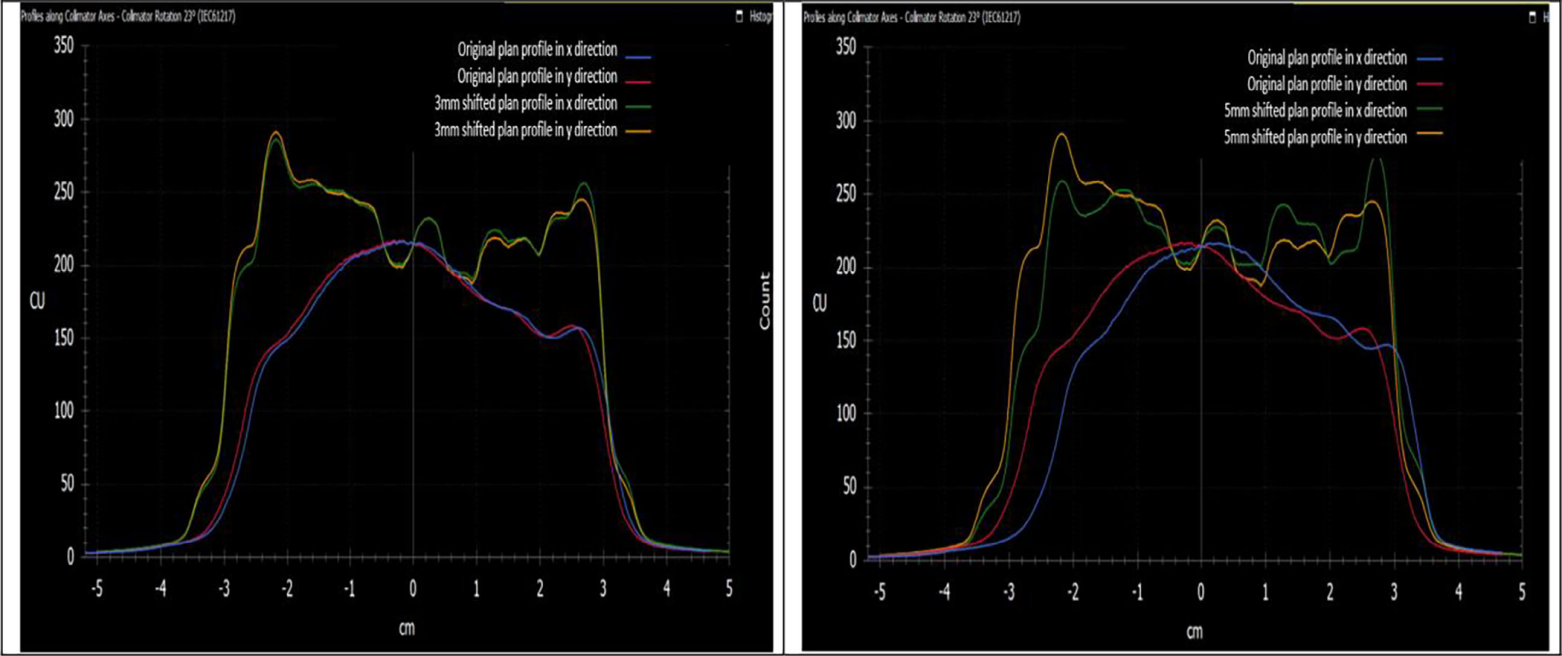
Shows the profile of the lung treatment plan shifted the MLC for each control point 3 mm in left figure and shifted the MLC for each control point 5 mm in Right figure.

**FIGURE 5:**
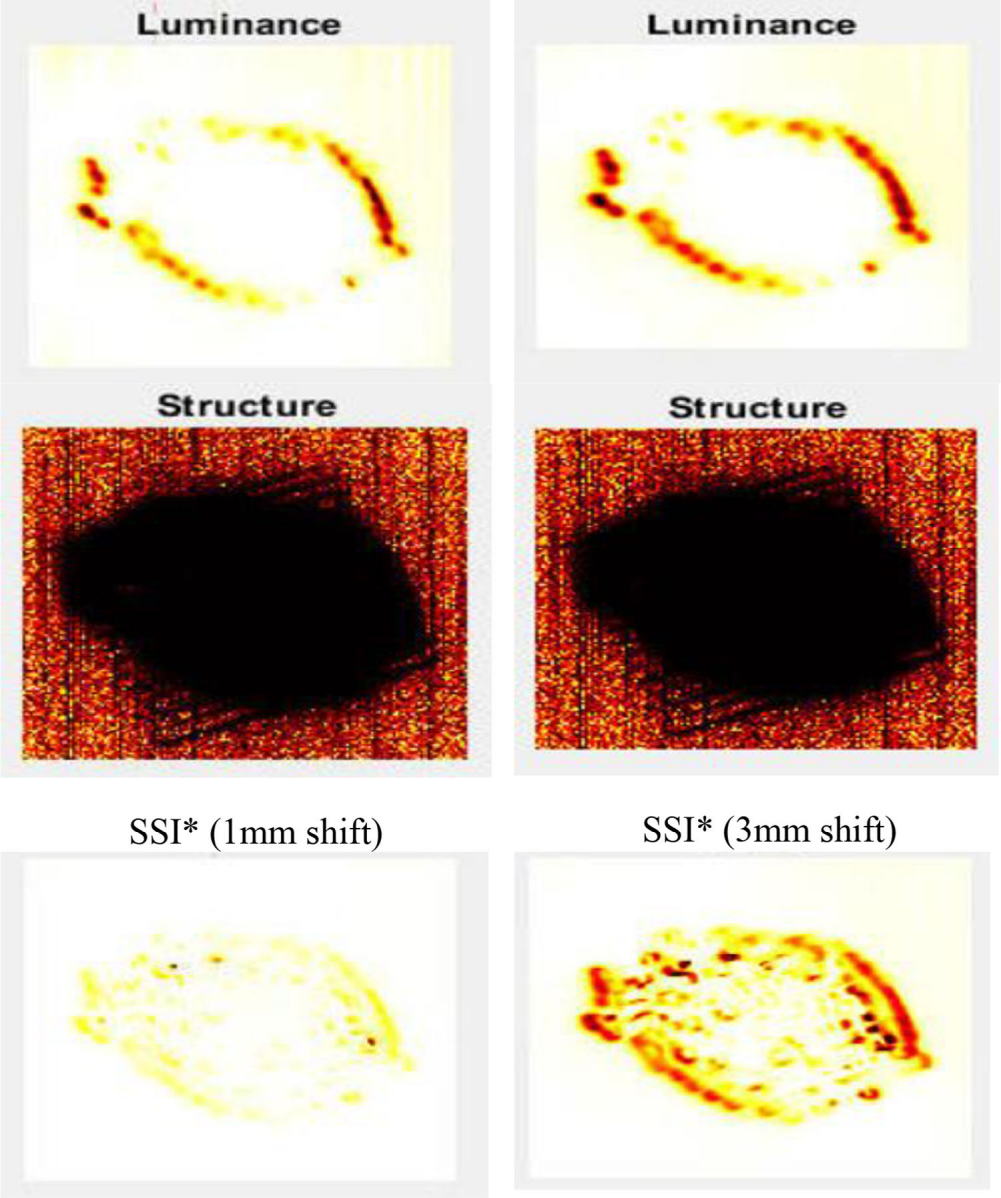
Luminance, Structure, and SSI* for 1 mm shift with 3 × 3 filter for the lung plan on the left and the Luminance, Structure, and SSI* for 3 mm shift with 3 × 3 filter on the right side.

We developed a VMAT Lung treatment plan utilizing a real patient's CT scan, wherein the MLC was systematically adjusted for each control point, shifted in increments of 0.5, 1, 3, and 5 mm.^35^, ^36^ Subsequently, both the original plan and the four variants, each with differing MLC shifts, were executed on a Truebeam machine. Figure 3 presents the fluence‐map of the original lung plan, while Figure 4 shows the profile of the lung treatment plan shifted 3 and 5 mm for each control point. In Figure 5, the visual representation illustrates the variance in the Luminance, Structure, and SSI* for the fluence‐map in Figure 3 for 1 mm and 3 mm shifts. The comparison is focused on the effects of a 1 mm shift with a 3 × 3 filter on the left side and a 3 mm shift with the same 3 × 3 filter on the right side.

A comparative analysis was conducted by evaluating the portal images of the shifted plan against the original plan, employing the Gamma index (3 mm/3% and 1 mm/3%) and SSI*. The detailed findings are presented in Table 6.

**TABLE 6:** MLC shift of a Lung treatment plan for patient‐specific QA.

Shift	0.5 mm	1 mm	3 mm	5 mm
Gamma index (3 mm/3%)	100%	100%	100%	69.5%
Gamma index (1 mm/3%)	100%	100%	100%	33.1%
l' (3 × 3)	0.997	0.996	0.974	0.948
*C_S* (3 × 3)	0.994	0.99	0.949	0.913
SSI* (3 × 3)	0.994	0.987	0.923	0.876
l' (5 × 5)	0.997	0.996	0.974	0.948
*C_S* (5 × 5)	0.995	0.987	0.933	0.888
SSI* (5 × 5)	0.993	0.984	0.914	0.857

## 4 DISCUSSION

Based on previous studies, a 1 mm MLC shift can result in a dose change of −2.1% for the PTV, 0.04% for the cord, −0.7% for the heart, and −0.07% for the lung in a VMAT plan.^37^ That means that Gamma test fails to detect around −10% dose change for PTV caused by the MLC shift deviation. On the contrary, the dose change can be controlled to stay under 6%. A 1 Gy decrease to the PTV might reduce the tumor control probability (TCP) by around 3.5%.^38^ For a prescription of 60 Gy, 10% decrease may potentially decrease TCP up to 21%. This deficiency can be attributed to the Distance to Agreement (DTA) limitations in the Gamma index calculation, where the DTA component remained below 1 even when the actual DTA distance was less than 3 mm.

Table 1 illustrates the limitations of the Gamma index test in detecting MLC shifts. With a 3 mm/3% criteria, the Gamma index test failed to identify shifts up to 5 mm, and with a more stringent 1 mm/3% criteria, it failed to detect shifts up to 3 mm. In contrast, the SSI* metric demonstrated better performance. The l' component of SSI* consistently dropped from 0.999 for 0.5 mm shift to 0.97 for 5 mm shift, indicating its effectiveness in detecting MLC position deviations. Additionally, the C_S component of SSI* decreased to 93% with a 2 mm shift, suggesting its efficiency in detecting MLC position deviations.

Similar observations were made in Table 2 regarding the detection of dose changes. The Gamma index test with a 3 mm/3% criteria failed to detect changes below 5%, and with a 1 mm/3% criteria, it failed to detect changes below 4%, once again due to the reliance on DTA. Conversely, the l' component in SSI* dropped to 0.947 with a 3% dose change, while the C_S component remained at 1 for all changes, indicating that the l' component is most effective in detecting dose changes.

**FIGURE 6:**
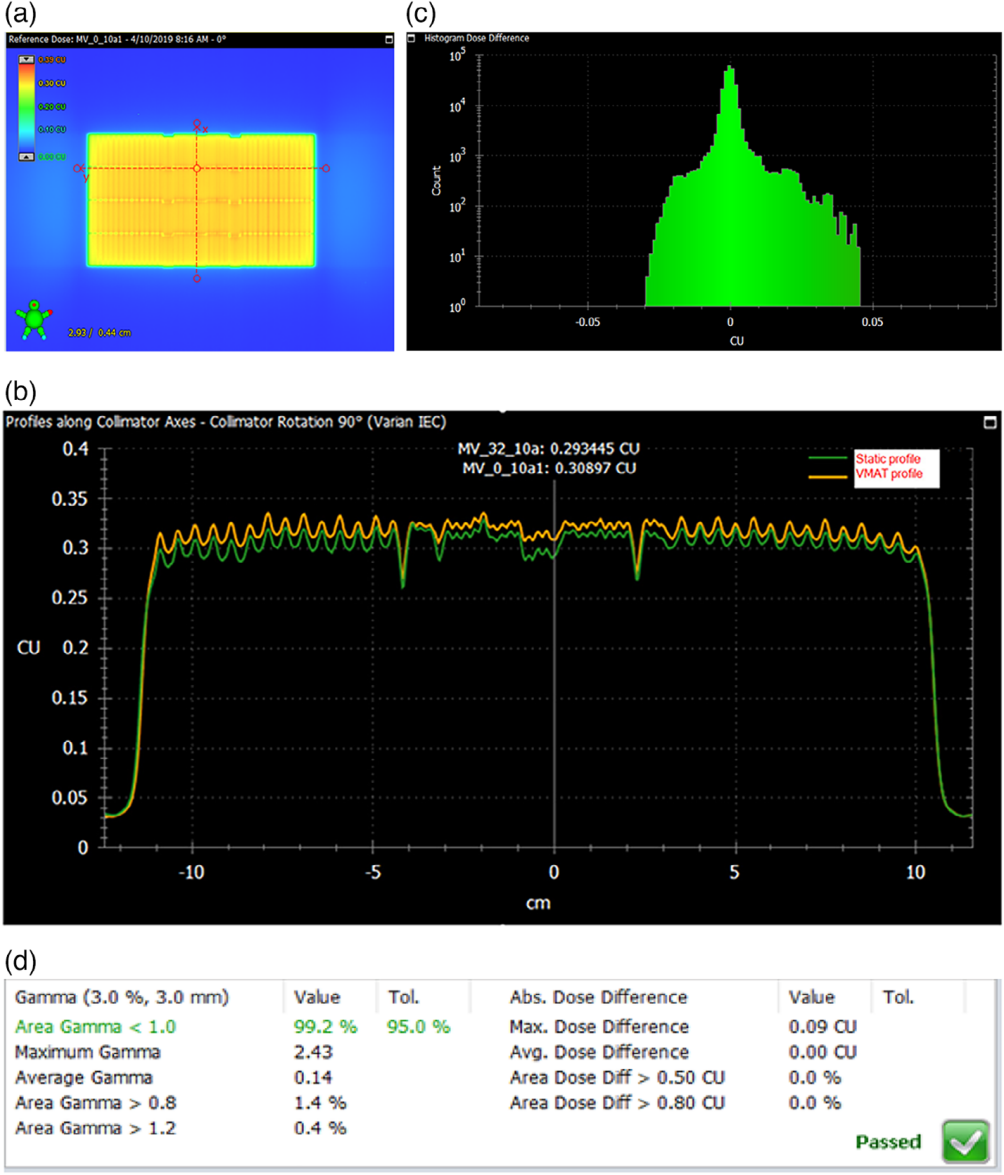
The Edge MLC QA week 6 (a) map‐Fluencee. (b) map‐fluence profiles along collimator axes, showing the difference between static and VMAT profile. (c) histogram dose difference between static and VMAT map‐fluence in Figure 6(a). (d) Gamma analysis evaluation report between static and VMAT image.

The results from the week 6 data point from the Edge is particularly interesting. Although this case passed with a 99.2 score using the Gamma index test (3 mm/3% criteria) and 98.4 score with the more stringent 1 mm/3% criteria, it failed with scores of 0.686 (3 × 3 sampling window) and 0.707 (5 × 5 sampling window) when evaluated using SSI*. In this case, the l' component in SSI* dropped to 0.7, indicating significant intensity (or dose) deviation, while the C_S component remained close to 1, signifying no significant MLC position deviation. Line profiles in Figure 6(a–d) further supported these findings, showing similar MLC positions but noticeable dose deviations of approximately 5% that went undetected by the Gamma index test.

The presented Edge QA in Table 3 serves as a concrete example, demonstrating the ability of SSI* to detect significant intensity (dose) deviations that were missed by the Gamma index test.

The findings from the investigation of the real lung patient VMAT plan in Section 3.4 indicate that SSI* demonstrated sensitivity in detecting MLC shifts at approximately 3 mm, while the Gamma test proved effective in identifying deviations at around a 5 mm MLC shift.

While the study's outcomes pertain specifically to Varian machines equipped with MLC log file capabilities, the suitability for Elekta Linac remains uncertain owing to the absence of corresponding data available for analysis.

Overall these results highlight the limitations of the Gamma index test and emphasize the superiority of the SSI* metric in detecting both MLC position deviations and dose changes.

## 5 CONCLUSIONS

The revised luminance component (l') in SSI* demonstrated improved sensitivity in detecting dose changes, while the contrast and structure components (C_S) in SSI* efficiently detect MLC position deviations compared to the conventional Gamma index test. As a result, SSI* shows potential superiority over the Gamma index test for multi‐leaf collimator (MLC) weekly quality assurance (QA). By incorporating SSI* into MLC QA protocols, we can benefit from its enhanced ability to identify dose variations and accurately detect deviations in MLC position. This can contribute to a more comprehensive and reliable assessment of treatment accuracy, ensuring high‐quality radiation therapy delivery.

## AUTHOR CONTRIBUTIONS

Hong Zhang: Involved in the methodology of the study; collected the data, developing algorithm; and performed the statistical analysis. Additionally; Hong Zhang contributed significantly to the writing of the manuscript. Baoshe Zhang: Responsible for data collection and helped in the interpretation of experimental results. Baoshe Zhang also contributed to the editing of the manuscript. Giovanni Lasio: Responsible for data collection; and data interpretation. Giovanni Lasio also reviewed the manuscript for clarity; grammar; and coherence. Shifeng Chen: data collection; and data interpretation. Shifeng Chen also critically reviewed the manuscript and provided valuable insights throughout the research process. Joubin Nasehi Tehrani: Involved in the initial conceptualization of the study, contributing significantly to the development of the study's methodology, developing essential codes and algorithms for evaluating the new method, the statistical analysis and the data collection. Joubin Nasehi Tehrani also supervised the project; and contributed to manuscript writing and editing. He also reviewed and edited the manuscript for clarity; grammar; and coherence. All authors have read and approved the final version of the manuscript.

## CONFLICT OF INTEREST STATEMENT

The authors declare no conflicts of interest.
